# Universal Strategy for Phase‐Selective Synthesis of Monolayer TMDs via Colloidal Chemistry

**DOI:** 10.1002/adma.74335

**Published:** 2026-07-25

**Authors:** Hoon Ju Lee, Weiguang Yang, Dae Young Park, Mrinal Kanti Kabiraz, Benoit Mahler, Hyeon Suk Shin

**Affiliations:** ^1^ Center For 2D Quantum Heterostructures Institute For Basic Science (IBS) Sungkyunkwan University (SKKU) Suwon Republic of Korea; ^2^ Department of Chemistry Ulsan National Institute of Science and Technology (UNIST) Ulsan Republic of Korea; ^3^ Department of Science Education Jeonju University Jeonju Republic of Korea; ^4^ Institut Lumière Matière UMR5306 CNRS Université Claude Bernard Lyon 1 Villeurbanne France; ^5^ Department of Chemistry Sungkyunkwan University (SKKU) Suwon Republic of Korea; ^6^ Department of Energy Sungkyunkwan University (SKKU) Suwon Republic of Korea

**Keywords:** 2H and 1T’ phases, colloidal chemistry, ligand engineering, monolayer transition metal dichalcogenides, phase engineering, phase‐selective synthesis

## Abstract

Colloidal synthesis of monolayer transition metal dichalcogenides (TMDs) remains challenging regarding uniform size, thickness, and crystalline phase. While significant progress has been made in phase engineering, a unified chemical strategy for on‐demand selection of both 2H and 1T′ phases across multiple TMD systems within a single chemical synthesis strategy has remained elusive. Here, we report a universal colloidal strategy for selective synthesis of both 2H and 1T′ phases across four representative TMDs — MoS_2_, MoSe_2_, WS_2_, and WSe_2_ — by tuning the metal‐to‐chalcogen precursor ratio ([M]:[X]) and ligand composition. We demonstrate that phase selection in W‐based TMDs is governed primarily by precursor stoichiometry: relatively lower chalcogen ratios favor the metastable 1T′ phase, while higher ratios stabilize the thermodynamic 2H phase. In contrast, Mo‐based TMDs remain locked in the 2H phase regardless of precursor ratios; however, the introduction of oleic acid into an oleylamine‐based ligand environment induces successful synthesis of 1T′ phase. This work represents, for the first time, a general approach for phase‐selective synthesis of all four TMD analogues using common precursors and ligands. These results establish a generalized design principle for phase‐selective colloidal synthesis of TMDs and provide a foundation for exploring phase‐dependent quantum phenomena and functional properties.

## 1 Introduction

Monolayer transition metal dichalcogenides (TMDs) have attracted intense interest due to their unique excitonic and electronic properties, originating from strong quantum confinement in the 2D limit, tunable electronic structures, and distinct phase‐dependent properties [1, 2, 3]. To harness these functionalities, precise control over the lateral dimension, thickness, and crystalline phase of these materials is essential. However, independent control of these parameters in bottom‐up colloidal synthesis remains significantly limited [4, 5].

TMDs can exist in either the semiconducting 2H phase or the metallic 1T/1T′ phases [4]. Since the electronic structure and physical properties diverge drastically depending on the crystalline phase, phase‐selective control during synthesis is a critical challenge [6, 7]. Despite progress, previous colloidal routes have largely focused either on the synthesis of a specific phase in a single TMD system (e.g., MoS_2_ or WS_2_, targeting 2H and/or 1T′) [8, 9, 10] or on achieving high‐purity 1T′ phases across diverse TMDs [11, 12] by relying on system‐dependent modifications. Consequently, the lack of a unifying synthetic principle has constrained the development of phase‐selective synthesis of TMDs and has led to inconsistent results across the literature, depending on precursor reactivity, ligand environment, chalcogen supply, nucleation kinetics, and reaction temperature. Although metal chlorides such as MoCl_5_ and WCl_6_ and fatty ligands such as oleylamine and oleic acid are frequently employed in TMD syntheses, their systematic and coordinated role in governing phase selectivity across diverse TMD systems remains underexplored.

In the case of Mo‐based TMDs (MoS_2_, MoSe_2_), colloidal synthesis almost exclusively yields the 2H phase [9, 13, 14]. To date, there have been a limited number of reports of selectively obtaining the metastable 1T′ phase during the synthesis stage [6, 11]. This is probably attributed to the relatively compact 4d orbitals of molybdenum, which are less effective in stabilizing the metal‐metal bonded distortions required for the 1T′ lattice and finally make the trigonal prismatic coordination of the 2H structure thermodynamically dominant [15, 16, 17]. Consequently, Mo‐based TMDs have faced inherent limitations in phase engineering and the development of metallic‐phase nanostructures. In contrast, the more extended 5d orbitals in W‐based TMDs (WS_2_, WSe_2_) facilitate Peierls‐like distortions, allowing the 1T′ phase to be more readily accessible [18, 19]. However, the synthesis of pure 2H monolayer W‐based TMDs has been extremely difficult. Previous reports have predominantly shown flower‐like aggregates, multi‐layered morphologies, or mixed phases [14, 20]. This is because the neutral basal plane of the 2H phase interacts weakly with amine‐based ligands, leading to vertical stacking and irreversible aggregation [8, 21].

Here, we report a universal colloidal synthesis strategy for selective synthesis of the 2H and 1T′ phases of four TMDs nanosheets (MoS_2_, MoSe_2_, WS_2_, and WSe_2_) by tuning the metal‐to‐chalcogen molar ratio and ligand composition. Based on a comprehensive analysis of previous reports, we utilized the most ubiquitous precursors (e.g., metal chlorides) and ligands (oleylamine and oleic acid) to establish a generalized protocol. We demonstrate that for W‐based TMDs, the metastable 1T′ phase is selectively synthesized under relatively lower chalcogen concentrations, whereas the thermodynamically stable 2H phase becomes dominant as the chalcogen ratio increases. In contrast, for Mo‐based TMDs, which inherently favor the 2H phase regardless of precursor ratios, we show that modulating the ligand composition‐specifically by introducing oleic acid‐enables the selective formation of the 1T' phase. Our findings provide a fundamental design principle for the phase‐controlled colloidal synthesis of TMDs and lay the groundwork for investigating phase‐dependent quantum phenomena and physical properties.

## 2 Results and Discussion

**FIGURE 1:**
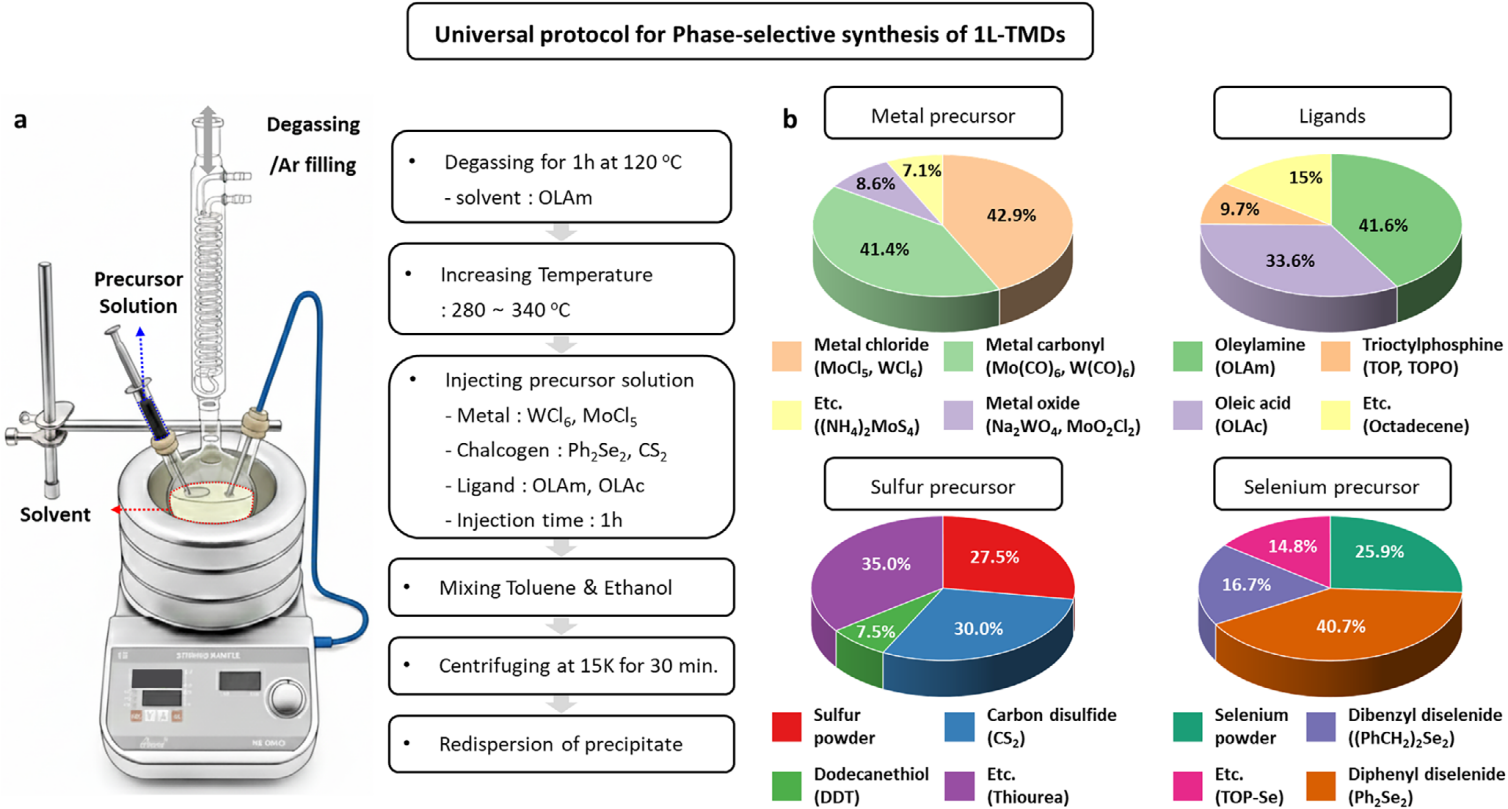
Experimental schematic and frequency of chemicals for TMD colloidal synthesis.

The right panel of Figure 1 summarizes the frequency of precursors and ligands used in 38 previously reported studies on TMD colloidal synthesis (see Supporting Information and Table S1). Metal chlorides (e.g., MoCl_5_, WCl_6_) were identified as the most common metal precursors, while sulfur powder, CS_2_, and DDT were widely used for sulfur sources, and Ph_2_Se_2_ was the predominant selenium precursor. Regarding ligands, oleylamine (OLAm) and oleic acid (OLAc) were the most frequently employed, playing critical roles in controlling the reaction medium and surface chemistry of the reaction environment. Based on this analysis, we established a universal protocol for the phase‐selective synthesis of four Mo‐ and W‐based monolayer TMD nanosheets (MoS_2_, MoSe_2_, WS_2_, and WSe_2_) using these widely used metal precursors (MoCl_5_ or WCl_6_), chalcogen precursors (Ph_2_Se_2_ or CS_2_), and ligand combinations (OLAm and OLAc) (Figure 1).

OLAm functions dualistically as both a surface ligand and a reaction medium (solvent), facilitating the dissociation and solubilization of precursors during the reaction [22]. In contrast, OLAc serves as a typical surface ligand, tuning the overall polarity and reactivity of the ligand environment through acid‐base interactions with OLAm [23, 24, 25, 26]. It is known that the primary interaction between OLAm and OLAc involves a rapid acid–base reaction, generating oleylammonium and oleate species [23, 27]. Although we cannot exclude the possibility of a condensation reaction between OLAm and OLAc to form N‐oleyloleamide and water at the elevated reaction temperature (∼300°C), we did not observe any macroscopic phase separation during the reaction, which would be expected if a substantial amount of water had been produced. All syntheses were conducted with a fixed total ligand volume of 5 mL. We systematically investigated the effects of the precursor molar ratio ([M]:[X]) and ligand composition (relative volume ratio of OLAm to OLAc, V_OLAc_/V_Total_) on the crystalline phase formation, while keeping other parameters such as precursor types and injection rates constant to minimize experimental variables. The particles obtained were purified using a toluene/ethanol mixture and redispersed in toluene for further analysis. The reaction temperatures ranged from 280 to 340°C depending on the specific TMD, but, within this optimized window, the temperature itself did not influence the resulting phase in our optimized conditions. Furthermore, extending the precursor injection time (reaction time) from the standard 1 h to 3 or 5 h did not affect the phase, lateral size, or layer number of the synthesized TMDs (Figure S1).

Table 1 summarizes the phase evolution of the synthesized TMDs as a function of the precursor molar ratio and ligand composition. The products were primarily monolayer nanosheets. The morphological assignments (i.e., monolayer vs. multilayer) presented in Table 1 reflect the statistically dominant layer number for each synthetic condition. Furthermore, the overall structural uniformity was rigorously verified by compiling both thickness and lateral size data from multiple HAADF‐STEM images acquired across randomly selected regions (see Figure S2 for lateral size distributions, and Figure S3 and Table S2 for thickness statistics). For W‐based TMDs (WS_2_ and WSe_2_), adjusting the [M]:[X] ratio revealed a distinct phase evolution. At relatively lower chalcogen loading, the monolayer 1T′ phase was preferentially synthesized. Conversely, increasing the chalcogen ratio favored the thermodynamically stable 2H phase, leading to multilayer stacking. Interestingly, for the 1T' W‐based TMDs synthesized under low chalcogen ratios, increasing the OLAc fraction in the ligand composition did not induce a phase change.

**TABLE 1:** Phase evolution of four TMD nanosheets (WS_2_, WSe_2_, MoS_2_, MoSe_2_) as a function of precursor ratio ([M]:[X], top row) and ligand composition (V_OLAc_/V_total_, bottom row).

Effect of [M]:[X] molar ratio	WS_2_			WSe_2_			MoS_2_			MoSe_2_		
	[M]:[X]	Morph.	Phase	[M]:[X]	Morph.	Phase	[M]:[X]	Morph.	Phase	[M]:[X]	Morph.	Phase
(Solvent/Ligand: OLAm)	1:5 ∼ 1:11^a^	Monolayer	1T`	1:2^c^	Monolayer	1T`	1:5^e^	Monolayer	2H	1:2^f^	Monolayer	2H
*1T`+2H = a physical mixture of individual 1T' and 2H nanosheets.	1:16 ∼ 1:20	Multilayer	1T`	1:3	Multilayer	1T`				1:4	Multilayer	2H
	1:21 ∼ 1:32	Multilayer	1T` major +2H	1:4	Multilayer	1T` major + 2H						
	1:60 ∼ 1:70^b^	Multilayer	1T` + 2H major	1:20^d^	Multilayer	1T` + 2H major	1:8	Multilayer	2H	1:20	Multilayer	2H

**Effect of OLAc volume fraction**	**WS_2_** **[M]:[X] = 1:5**			**WS_2_** **[M]:[X] = 1:64**			**WSe_2_** **[M]:[X] = 1:3**			**WSe_2_** **[M]:[X] = 1:20**			**MoS_2_** **[M]:[X] = 1:5**			**MoSe_2_** **[M]:[X] = 1:2**		
	**V_OLAc_/V_total_**	**Morph**.	**Phase**	**V_OLAc_/V_total_**	**Morph**.	**Phase**	**V_OLAc_/V_total_**	**Morph**.	**Phase**	**V_OLAc_/V_total_**	**Morph**.	**Phase**	**V_OLAc_/V_total_**	**Morph**.	**Phase**	**V_OLAc_/V_total_**	**Morph**.	**Phase**
V_OLAc_/V_total_ V_total_ = V_OLAc_ + V_OLAm_ = 5 mL	0	Monolayer	1T’	0	Multilayer	1T`+2H major	0	Multilayer	1T`	0	Multilayer	1T` major+2H	0	Monolayer	2H	0	Monolayer	2H
	0.1	Monolayer	1T’				0.1	Multilayer	1T`				0.1	Monolayer	1T`major + 2H			
	0.2	Monolayer	1T’				0.2	Multilayer	1T`				0.2	Monolayer	1T`	0.2	Monolayer	1T`+2H major
	0.3	Monolayer	1T’	0.3	Multilayer	1T`	0.3	Multilayer	1T`	0.3	Multilayer	1T`	0.3	Monolayer	1T`	0.3	Monolayer	1T`major +2H
													0.4^g^	Monolayer	1T`	0.4^h^	Monolayer	1T`

TMDs were synthesized under the conditions summarized in the table, and representative TEM images are shown in the main text corresponding to.

^a^ Figure 2a,

^b^ Figure 2b,

^c^ Figure 2c,

^d^ Figure 2d,

^e^ Figure 3a,

^f^ Figure 3c,

^g^ Figure 3b, and

^h^ Figure 3d

In the case of Mo‐based TMDs (MoS_2_ and MoSe_2_), the thermodynamically stable 2H phase was obtained regardless of the [M]:[X] ratio. Similar to the W‐series, increasing the chalcogen ratio led to the stacking of monolayers into multilayers. However, unlike the [M]:[X] ratio dependence observed in W‐based TMDs, the precursor ratio alone could not induce a phase transition in Mo‐based TMDs [28]. Remarkably, modifying the ligand composition by increasing the OLAc fraction successfully selectively synthesized the 1T' phase from the 2H‐forming conditions. As noted, while the 1T' phase of W‐based TMDs was maintained upon OLAc addition, the 2H‐dominant W‐based TMDs (formed under high chalcogen conditions) also transformed into the 1T' phase when OLAc was added. Consequently, the addition of OLAc consistently promoted the formation of the 1T' phase for both 2H‐MoX_2_ and 2H‐WX_2_.

First, we discuss why the simplest synthesis condition (low [M]:[X] ratio with pure OLAm; top row of Table 1) yields the 1T' phase for WX_2_ but the 2H phase for MoX_2_. This discrepancy implies a significant difference in reactivity between W and Mo. The energy difference between the 2H and 1T' phases is known to be much larger for Mo‐based TMDs than for their W‐based counterparts, making the 1T' phase of Mo significantly more unstable and difficult to access [17, 18, 29]. Thus, the 1T′ phase can be accessed in WS_2_ under appropriately tuned conditions. Furthermore, the relatively compact 4d orbitals of Mo are spatially localized, favoring the trigonal prismatic (2H) coordination over the distorted structures formed by metal‐metal bonding. In contrast, the more extended 5d orbitals of W facilitate the formation of W–W zigzag chains, providing a stabilization mechanism for the Peierls‐type lattice distortion characteristic of the 1T' phase [15, 16, 17]. Based on these fundamental differences, we discuss the representative results of phase‐selective synthesis for 1T'/2H WX_2_ and 2H/1T' MoX_2_ in the following sections (shaded regions in Table 1).

**FIGURE 2:**
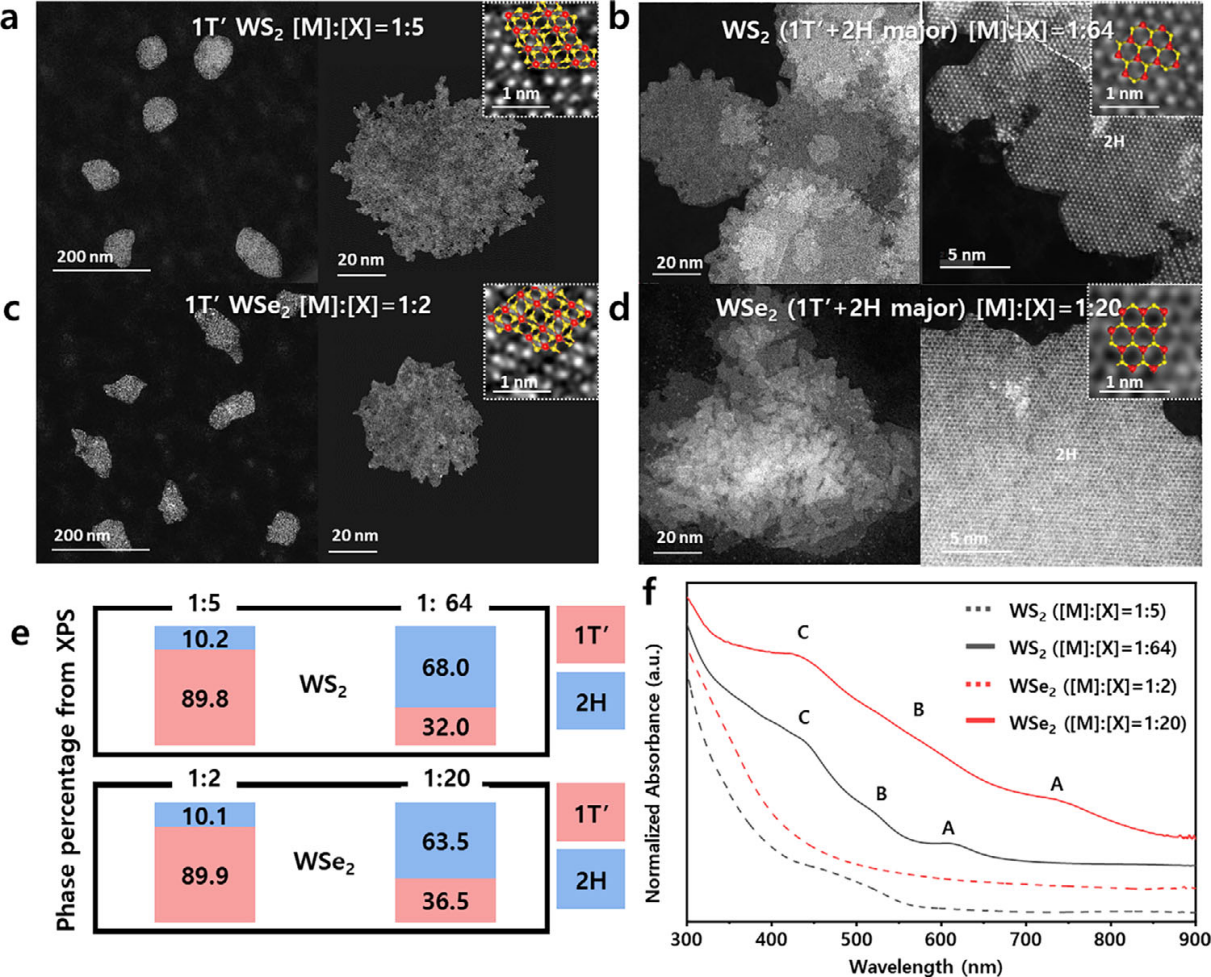
Phase‐selective synthesis of W based TMDs with the variation of [M]:[X] precursor ratio. HAADF‐STEM images of a and b) WS_2_ and c and d) WSe_2_ synthesized with varying [M]:[X] precursor ratios an under identical ligand conditions (V_OLAc_/V_total_ = 0). e) Quantitative phase analysis from X‐ray photoelectric spectroscopy (XPS). f) UV–Vis absorption spectra of 1T′ and 2H phases in WS_2_ and WSe_2_. The insets in a and c show atomic models of the 1T′ phase, whereas the insets in b and d show atomic models of the 2H phase.

Figure 2 presents representative HAADF‐STEM images of WS_2_ and WSe_2_ nanosheets synthesized with varying precursor ratios and ligand compositions, confirming their crystalline phases. As summarized in Table 1, a relatively lower chalcogen ratio (higher metal supply) favors the formation of the 1T' phase in both WS_2_ and WSe_2_ systems, whereas a relatively higher chalcogen ratio promotes the 2H phase. For WS_2_ (Figure 2a), a pure 1T' phase is observed at a relatively low chalcogen condition ([M]:[X] = 1:5). At [M]:[X] = 1:32, a mixed phase containing both 1T' and 2H nanosheets is observed (Figure S4a), with the 1T' phase remaining dominant. Further increasing the chalcogen supply to [M]:[X] = 1:64 results in the dominance of 2H nanosheets (Figure 2b). A similar trend is observed for WSe_2_; a monolayer 1T' phase is clearly formed at [M]:[X] = 1:2 (Figure 2c), while a mixed phase appears at [M]:[X] = 1:4 (Figure S4b). Under excess chalcogen conditions such as [M]:[X] = 1:20, the 2H phase becomes dominant (Figure 2d). The phase controlled effect of [M]:[X] ratio in WS_2_ and WSe_2_ is also confirmed through X‐ray photoelectron spectroscopy (XPS) (Figures S5 and S6), UV‐Vis absorption, and Raman scattering (Figure S7). The phase purities of WS_2_ and WSe_2_ synthesized with low [M]:[X] ratios, 1:5 for WS_2_ and 1:2 for WSe_2_, are approximately 90% of 1T’ phase (Figure 2e). So, the absorption spectra show the monotonic increase of absorption as decreasing the wavelength of incident light (Figure 2f). In the case of high [X] ratio, the percentage of 2H phase is increased around 65% and excitonic features of TMDs are appeared in absorption spectra. The representative vibration modes denoted A_1g_ (out‐of‐plane) and E^1^_2g_ (in‐plane) are observed in high [X] ratio samples. Crucially, the Raman spectra of W‐based TMDs synthesized at relatively lower chalcogen loading display only the J modes characteristic of the 1T′ phase, without any distinct 2H phase signatures (Figure S7a,b). This result corroborates the high 1T′ phase purity (∼90%) quantified by XPS [11, 30].

We propose two mechanisms to explain the stabilization of the 2H phase at relatively high chalcogen ratios. First, an abundance of chalcogen ligands (S^2–^ or Se^2–^) restricts the spatial and electronic freedom required for the formation of direct metal‐metal bonds (W‐W zigzag chains). Instead, the formation of a metal‐chalcogen‐metal network is favored, stabilizing the trigonal prismatic coordination (2H phase). Second, a sufficient chalcogen supply suppresses the formation of chalcogen vacancies during growth, thereby reducing local lattice distortions and electron density inhomogeneities. This prevents the Peierls‐type distortion necessary for the transition to the 1T' phase and promotes the growth of the more ordered 2H crystal structure [31, 32].

**FIGURE 3:**
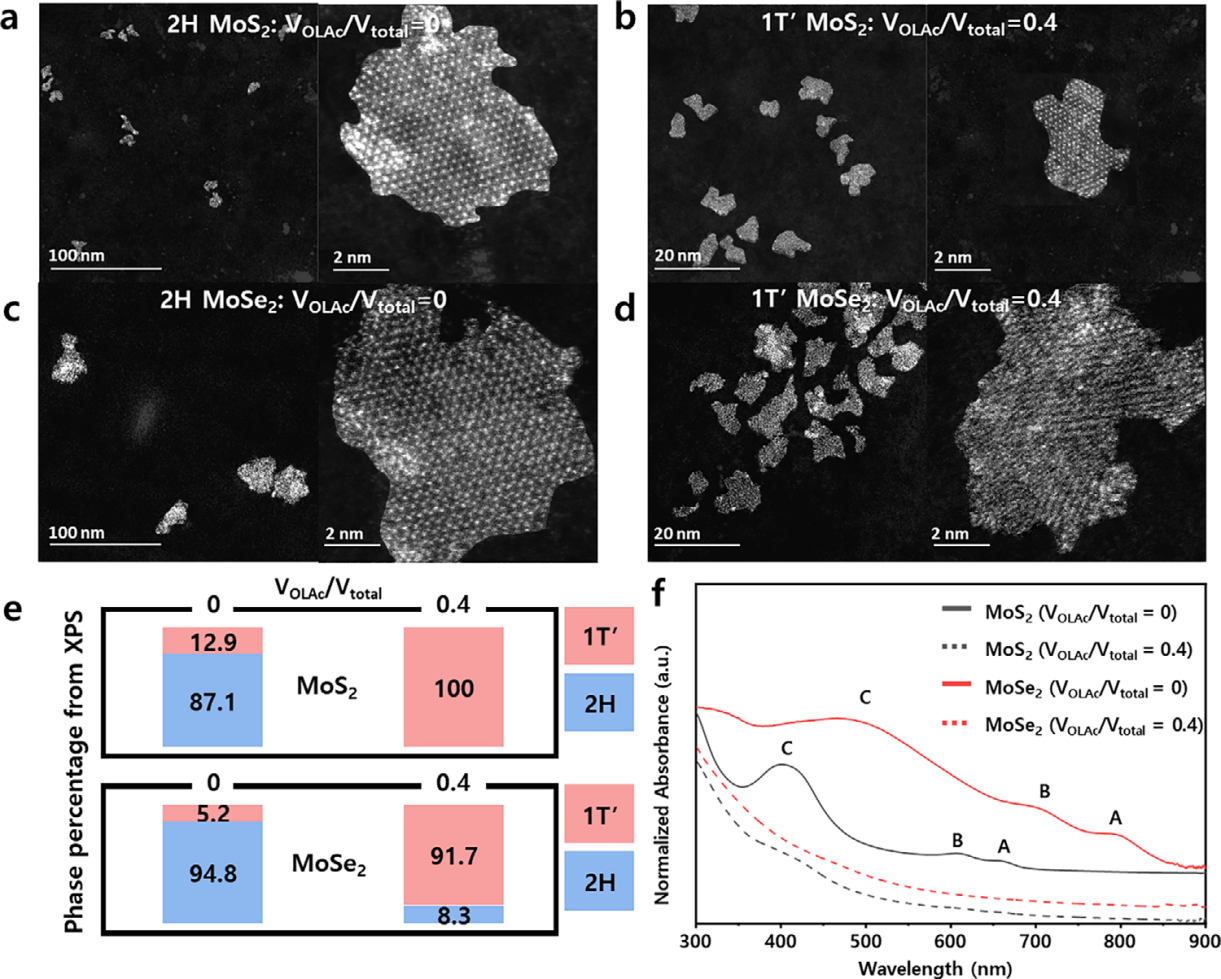
Phase‐selective synthesis of Mo based TMDs with the variation of ligand volume ratios. HAADF‐STEM images of a and b) MoS_2_ and c and d) MoSe_2_ synthesized with varying volume ratios between oleylamine and oleic acid under identical [M]:[X] ratio ([Mo]:[X] = 1:5 for MoS_2_, 1:2 for MoSe_2_). e) Quantitative phase analysis from XPS. f) UV–Vis absorption spectra of 1T′ and 2H phases in MoS_2_ and MoSe_2_.

As shown in Table 1, MoS_2_ and MoSe_2_ nanosheets synthesized using only OLAm exhibited the 2H phase regardless of the [Mo]:[X] ratio. Even under relatively low chalcogen conditions, the 2H phase persisted, indicating that precursor ratio control alone is insufficient to induce phase transition in Mo‐based TMDs. Increasing the chalcogen ratio also led to the stacking of monolayers into multilayers. This tendency is consistent with previous reports showing that MoS_2_ predominantly retains the 2H phase under a wide range of ligand and precursor conditions [9, 33]. However, altering the ligand composition by adding OLAc successfully induced a phase transition from 2H to 1T' (Figure 3).

For MoS_2_, a pure 2H phase is observed under OLAc‐free conditions (V_OLAc_/V_Total_ = 0) (Figure 3a). The introduction of OLAc (V_OLAc_/V_Total_ = 0.1) leads to the formation of a mixed phase (Figure S8a), and a further increase to V_OLAc_/V_Total_ = 0.4 results in a predominantly 1T' phase (Figure 3b). A similar trend is observed for MoSe_2_; the thermodynamically stable 2H phase forms without OLAc (Figure 3c), while a mixed phase appears at a ratio of 0.2 (Figure S8b), and a pure monolayer 1T' phase is synthesized at V_OLAc_/V_Total_ = 0.4 (Figure 3d). The phase ratio calculated from XPS demonstrates the effect of OLAc for the phase selective synthesis of Mo based TMDs. In the OLAc‐free condition, 2H phase of MoS_2_ and MoSe_2_ is reached to 87.1% and 94.8%, respectively (Figure 3e). In the case of V_OLAc_/V_Total_ = 0.4, the percentage of 2H phase for MoSe_2_ and MoS_2_ is dramatically reduced to 8.3% and 0%. The change of dominant phase is also observed in absorption spectra (Figure 3f). Mo based TMDs synthesized in OLAc‐free condition clearly appears A, B, and C exciton peaks, indicating semiconducting 2H phase. However, the spectra of MoS_2_ and MoSe_2_ synthesized with OLAC addition (V_OLAc_/V_Total_ = 0.4) are changed to typical metallic absorption spectra, indicating absence of 2H phase. Besides, the Raman scattering of Mo based TMDs are well presented the conversion of phase according to V_OLAc_/V_Total_ (Figure S7). At the high V_OLAc_/V_Total_, significant Raman intensities of J modes are observed both MoS_2_ and MoSe_2_, however, only A_1g_ and E_12g_ vibrational modes are existed from Mo based TMDs synthesized in the OLAc‐free condition (Figure S7c,d) [11, 30].

This ligand‐induced phase control was found to be universal, as the addition of OLAc also converted the 2H‐dominant W‐based TMDs (formed under high chalcogen conditions) into the 1T' phase (Figure S9). These results demonstrate that phase control, which was unachievable solely by adjusting the metal‐to‐chalcogen ratio in Mo‐based systems, can be effectively realized by modulating the ligand environment. Although the exact contribution of the complex ligand composition remains to be fully elucidated, we postulate that the oleate groups from OLAc may act as electron donors to the TMD surface, thereby stabilizing the electron‐rich 1T' phase.

We propose the following mechanism for phase‐selective TMD synthesis based on control of the precursor ratio ([M]:[X]) and ligand composition described above. In the case of the W‐series (WS_2_, WSe_2_), we observed that a high [M]:[X] ratio (metal excess) leads to a rapid increase in supersaturation within the solution, thereby triggering burst nucleation [34]. The high‐density initial clusters formed during this process possess the high chemical potential energy required to stabilize the distorted lattice of the 1T' phase. Conversely, a lower ratio maintains low supersaturation, inducing slow equilibrium growth that results in the formation of the thermodynamically stable 2H phase. OLAc reacts with metal chlorides to form metal‐oleate complexes. These complexes exhibit higher thermal stability than the original chloride precursors, effectively slowing the decomposition rate and lowering the reduction potential of metal ions [23]. This controlled precursor reactivity suppresses rapid and disordered growth, providing a favorable chemical environment for the stable formation of the metastable 1T' phase. Compared to W, Mo possesses more localized 4d orbitals, resulting in a significantly higher energy barrier between the 2H and 1T' phases. Our experimental results indicate that, unlike the W‐series, the Mo‐series remains locked in the 2H phase when only the precursor ratio (nucleation kinetics) is adjusted. Therefore, to induce the 1T' phase in Mo‐based TMDs, additional electronic stabilization, achieved through electron donation and the formation of precursor complexes via OA introduction, is essential.

**FIGURE 4:**
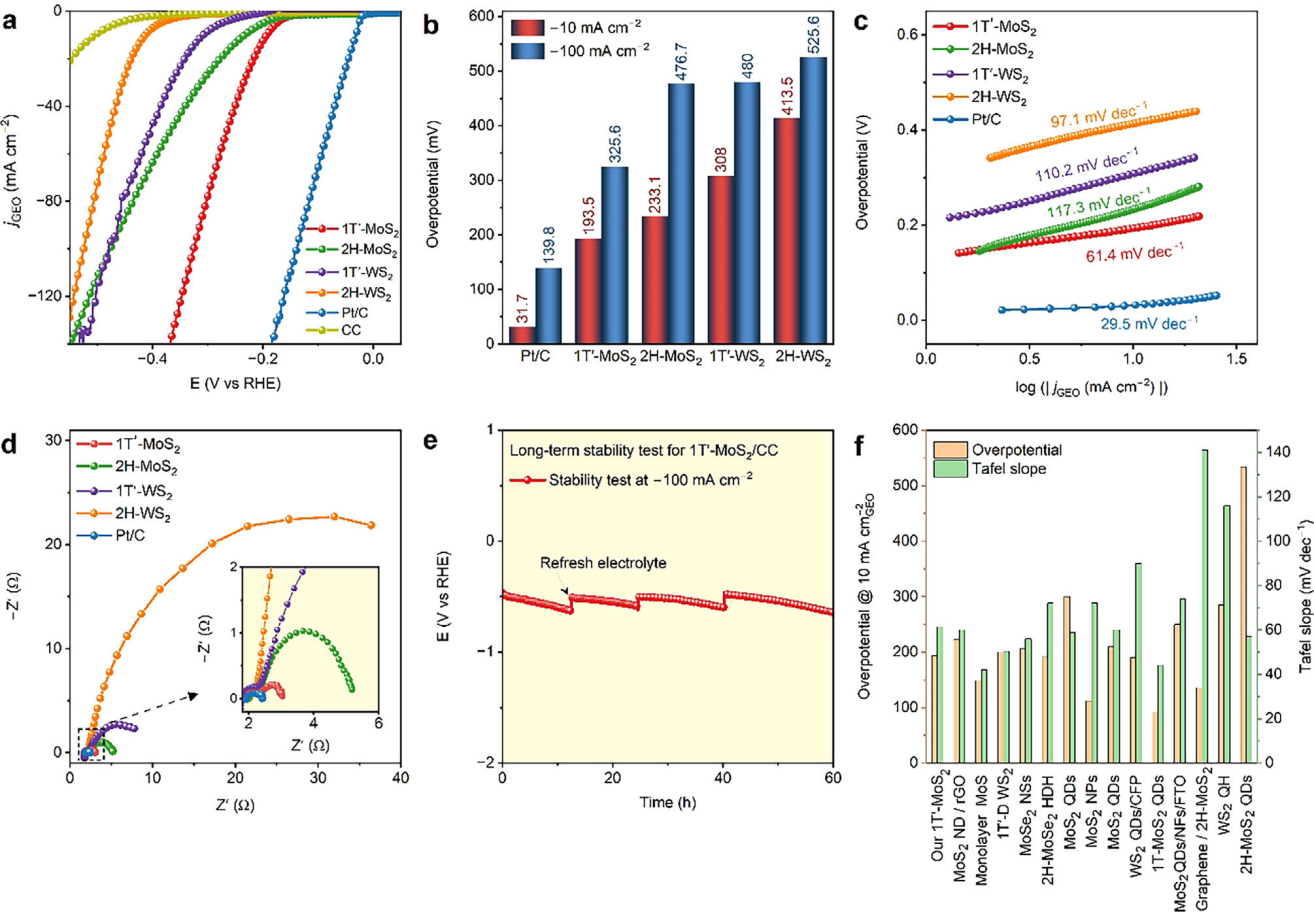
Electrocatalytic hydrogen evolution reaction (HER) performance of the synthesized sulfide TMD catalysts in Ar‐saturated 0.5 M H_2_SO_4_. a) Polarization curves of 1T′‐MoS_2_, 2H‐MoS_2_, 1T′‐WS_2_, 2H‐WS_2_, and commercial Pt/C. b) Corresponding overpotentials at a current density of −10 and −100 mA cm^−2^. c) Tafel plots derived from the polarization curves. d) Nyquist plot at 300 mV overpotential. e) Chronopotentiometric stability test of 1T′‐MoS_2_ at a constant current density of −100 mA cm^−2^. f) Comparison of HER performance with representative phase selective TMD‐based electrocatalysts reported in the literature.

To demonstrate the practical utility of our materials, we performed electrocatalytic experiments for the hydrogen evolution reaction (HER). The HER activity of the synthesized sulfide catalysts supported on carbon cloth (CC) was assessed in Ar‐saturated 0.5 M H_2_SO_4_ using a standard three‐electrode configuration. As shown in Figure 4a,b, 1T′ MoS_2_ exhibits the highest activity, requiring an overpotential of 193.5 mV to reach 10 mA cm^−2^, outperforming 2H MoS_2_ (233.1 mV), 1T′ WS_2_ (308 mV), and 2H WS_2_ (413.5 mV), while Pt/C delivers 31.7 mV as a benchmark. Tafel analysis (Figure 4c) reveals a slope of 61.4 mV dec^−1^ for 1T ‐MoS_2_, indicative of a Volmer–Heyrovsky mechanism, whereas higher slopes for the other catalysts reflect slower kinetics. Electrochemical impedance spectroscopy (Figure 4d) further confirms that 1T′ MoS_2_ exhibits the lowest charge‐transfer resistance, indicating enhanced interfacial electron transport. Furthermore, the 1T′ MoS_2_ catalyst demonstrated excellent long‐term stability at ‐100 mA cm^−2^ (Figure 4e), maintaining stable operation over 60 h with negligible potential variation (Figure S10). Benchmarking our results against other representative TMD electrocatalysts (Figure 4f and Table S3) indicates that our ligand‐capped 1T′ TMDs without any post‐treatment exhibit performance comparable to those produced by other specialized methods. These results demonstrate that the ligand‐capped TMDs synthesized via our universal strategy exhibit high overall quality and functional robustness, performing on par with those produced by other established methods. This indicates that our generalized approach yields properly stabilized and active TMD nanosheets without compromising their intrinsic catalytic potential.

## 3 Conclusion

In summary, we established a universal colloidal strategy for phase‐selective synthesis of monolayer transition metal dichalcogenides by systematically controlling precursor stoichiometry and ligand composition. Precise tuning of the metal‐to‐chalcogen ratio and ligand environment enables selective access to both 2H and 1T′ phases across MoS_2_, MoSe_2_, WS_2_, and WSe_2_. We demonstrate that phase selection in W‐based TMDs is predominantly governed by precursor stoichiometry, whereas Mo‐based TMDs intrinsically favor the 2H phase over a wide range of precursor ratios. Notably, introducing oleic acid into the ligand environment robustly induces a 2H‐to‐1T′ phase transition in both Mo‐ and W‐based systems, thereby overcoming the intrinsic phase limitation of Mo‐based TMDs and enabling the first colloidal synthesis of monolayer 1T′ MoX_2_ (X = S, Se). These results demonstrate how precursor stoichiometry and ligand‐mediated stabilization together influence phase evolution in colloidal TMD synthesis, in a manner consistent with established trends in phase energetics. The generality of this strategy provides a practical design guideline for phase‐engineered TMD nanostructures and opens new opportunities for exploring phase‐dependent electronic, catalytic, and quantum phenomena.

## 4 Experimental Section

### 4.1 Chemicals

All reagents were used as received without further purification. Molybdenum(V) chloride (MoCl_5_, 99.99%, trace metals basis), tungsten(VI) chloride (WCl_6_, 99.99%, trace metals basis), oleylamine (OLAm, technical grade, 70%), and oleic acid (OLAc, technical grade, 90%) were purchased from Sigma‐Aldrich. Carbon disulfide (CS_2_, ACS reagent, 99.9%) and diphenyl diselenide (Ph_2_Se_2_, 98%) were used as sulfur and selenium precursors, respectively. Toluene (99.5%) and ethanol (anhydrous, 99.9%) from Samchun were used for purification.

### 4.2 Synthesis of MoS_2_

MoS_2_ was synthesized using a hot‐injection method under an inert atmosphere. A 50 mL three‐necked round‐bottom flask equipped with a magnetic stir bar, thermocouple, and reflux condenser was placed on a heating mantle (see Figure 1). One port was sealed with a rubber septum for precursor injection. Oleylamine (5 mL) was loaded into the flask and degassed at 120°C under vacuum for 1 h. After degassing, Ar gas was flowed for 10 min to remove residual oxygen, and the temperature was ramped to the desired reaction temperature (280–340°C) under Ar atmosphere.

In a glovebox, MoCl_5_ (0.126 mmol) was dissolved in a 5 mL mixture of OLAc and OLAm at 50°C. The volume ratio of OLAc/OLAm was adjusted depending on the experimental condition, while the total volume remained fixed at 5 mL. CS_2_ was added to the metal precursor solution according to the desired [Mo]:[S] molar ratio. The resulting precursor solution was gently vortexed to prevent gelation and loaded into a syringe for injection.

Upon reaching the target reaction temperature, the precursor solution was injected into the reaction flask at a rate of 5 mL h^−^^1^ for 1 h. After injection, the flask was removed from the heating mantle and allowed to cool to room temperature under stirring.

The resulting colloidal dispersion was purified by adding 10 mL of toluene and 5 mL of ethanol, followed by vortexing and centrifugation at 15,000 rpm for 15 min. The supernatant was discarded, and the precipitate was redispersed in 20 mL of toluene.

### 4.3 Synthesis of WS_2_, MoSe_2_, and WSe_2_

For WS_2_ synthesis, WCl_6_ (0.126 mmol) was used as the metal precursor. For MoSe_2_ and WSe_2_, Ph_2_Se_2_ was directly dissolved with MoCl_5_ or WCl_6_ in the ligand mixture following the desired [M]:[Se] molar ratio. The subsequent procedures were identical to those described for MoS_2_.

### 4.4 Electrochemical Measurement

Electrochemical measurements were conducted using a VMP3 electrochemical workstation (Bio‐Logic Science Instruments, France) in a typical three‐electrode configuration, with a catalyst‐coated carbon cloth (CC) as the working electrode, a graphite rod as the counter electrode, and an Ag/AgCl (3 M KCl) electrode as the reference electrode. The reference electrode was calibrated against a reversible hydrogen electrode (RHE) in 0.5 M H_2_SO_4_ using two Pt plates as the working and counter electrodes, where H_2_ was generated in situ during cyclic voltammetry, and all potentials were converted to the RHE scale. The catalyst ink was prepared by dispersing 5 mg of catalyst in a mixed solvent containing 500 µL ethanol, 500 µL deionized (DI) water, and 20 µL of 5 wt% Nafion solution, followed by ultrasonication for 60 min to obtain a homogeneous suspension. The ink was drop‐cast onto CC (loading: 1.0 mg cm^−2^) and dried under oven. The electrodes were activated by cyclic voltammetry at a scan rate of 100 mV s^−1^ prior to measurements. The electrolyte (0.5 M H_2_SO_4_) was purged with Ar for at least 20 min before measurements. Linear sweep voltammetry was conducted at a scan rate of 5 mV s^−1^ with 85% iR compensation. Tafel slopes were derived from polarization curves and electrochemical impedance spectroscopy was performed over a frequency range of 100 kHz to 0.1 Hz with an amplitude of 5 mV. Long‐term stability was evaluated by chronopotentiometric measurements at a constant current density of 100 mA cm^−2^.

### 4.5 Characterizations

The as‐synthesized TMDs were comprehensively characterized to evaluate their composition, structure, and optical properties. For most characterizations, purified TMD colloidal dispersions were drop‐cast onto appropriate substrates. For UV–vis measurements, the dispersions were diluted with toluene and measured in a quartz cuvette.

X‐ray photoelectron spectroscopy (XPS, K‐Alpha, Thermo Scientific, USA), equipped with a monochromatic Al Kα X‐ray source, was employed to determine the chemical composition and valence states of the elements. All binding energies were calibrated using the adventitious C 1s reference peak at 284.8 eV.

High‐angle annular dark‐field scanning transmission electron microscopy (HAADF‐STEM, JEOL JEM‐ARM200F) was used to analyze the crystal structure, layer number, and lateral size of the TMD nanosheets. To ensure the statistical reliability of the morphological analysis, HAADF‐STEM imaging was performed on numerous randomly selected regions across the TEM grids, and the layer numbers of individual nanosheets were systematically counted to construct the thickness distribution profiles. The samples were prepared by drop‐casting diluted dispersions onto ultrathin carbon‐coated copper grids. The microscope was operated at an accelerating voltage of 80 kV to minimize electron beam‐induced damage to the ultrathin nanosheets.

Raman spectroscopy (alpha300, WITec, Germany) was performed to identify the crystal phases of the TMDs. The spectra were acquired using a 532 nm laser excitation. To prevent laser‐induced local heating and unintended phase transition (e.g., from 1T′ to 2H phase) during measurement, the laser power was maintained below 1 mW.

In addition, UV–vis absorption spectroscopy (JASCO V‐670, Japan) was carried out using a quartz cuvette to probe the optical absorption behavior of the TMD nanosheets dispersed in toluene. The spectra were recorded over a wavelength range of 300–900 nm with a scan rate of 200 nm min^−^^1^.

## Conflicts of Interest

The authors declare no conflicts of interest.

## Supporting information




**Supporting File**: adma74335‐sup‐0001‐SuppMat.docx.

## Data Availability

The data that support the findings of this study are available from the corresponding author upon reasonable request.
