# Supplementary material for: Universal Strategy for Phase‐Selective Synthesis of Monolayer TMDs via Colloidal Chemistry
Source: Adv Mater. 2026 Jul 25;38(53):e74335. doi: 10.1002/adma.74335 (PMC13591648; doi:10.1002/adma.74335)
Supplement: Supplementary file 1 [file ADMA-38-e74335-s001.docx]

Supporting Information

Universal Strategy for Phase-Selective Synthesis of Monolayer TMDs via Colloidal Chemistry

Hoon Ju Lee, Weiguang Yang, Dae Young Park, Mrinal Kanti Kabiraz, Benoit Mahler and Hyeon Suk Shin*

Keywords: Monolayer transition metal dichalcogenides, Phase-selective synthesis, Colloidal chemistry, 2H and 1T′ phases, Ligand engineering, Phase engineering

**Previous literature survey**

For checking the type and frequency of chemicals in TMDs colloidal synthesis, we used to google scholar site with “TMDs colloidal synthesis” as a key word. The search results were sorted by relevance and we chose 38 papers, for representative TMDs (MoS_2_, MoSe_2_, WS_2_, WSe_2_, totally 53) which we studied. The chemicals have been categorized to metal precursor, chalcogen precursor, and ligands. The summary table is shown below as Table S1.

**Table S1.** The summary for the types of chemicals used in previous studies on TMDs colloidal synthesis

| **TMDs** | **Metal**  **precursor** | **Chalcogen**  **precursor** | **Ligand 1** | **Ligand 2** | **Ligand 3** | **Phase** | **Phase Control Method** | **Universality** | **Ref.** |
| --- | --- | --- | --- | --- | --- | --- | --- | --- | --- |
| MoS_2_ | MoCl_5_ | CS_2_ | Ligand-free synthesis in Formamide  (Stabilized by inorganic ions, S²⁻) | | | 2H | - | X  (2H-MoS_2_, WS_2_) | ACS Appl. Mater. Interfaces 2020, 12, 13148−13155 |
|  |  | (NH_4_)_2_S |  |  |  |  |  |  |  |
| MoS_2_ | MoCl_5_ | Sulfur | Oleylamine | Octadecene |  | 2H | - | X  (2H-MoS_2_, MoSe_2_) | ACS Appl. Nano Mater. 2022, 5, 10311−10320 |
| MoS_2_ | (NH_4_)_2_MoS_4_ |  | Oleylamine | Oleic acid |  | 2H |  | X  (Only 2H MoS_2_) | ACS Appl. Nano Mater. 2024, 7, 163−169 |
| MoS_2_ | MoCl_5_ | Na_2_S | Oleylamine | Octadecene |  | 2H | - | X  (2H-MoS_2_, MoSe_2_) | ACS Appl. Mater. Interfaces 2024, 16, 36315 |
| MoS_2_ | MoCl_5_ | Na_2_S | Ligand-free synthesis in Formamide  (Stabilized by inorganic ions, S²⁻) | | | 2H | - | X  (Only 2H-MoS_2_) | ACS Appl. Nano Mater. 2023, 6, 9475-9483 |
| MoS_2_ | (NH_4_)_2_MoS_4_ |  | Oleylamine | Oleic acid | Octadecene | 2H | - | X  (Only 2H-MoS_2_) | Appl. Phys. Lett. 120, 253502 (2022) |
| MoS_2_ | (NH_4_)_2_MoS_4_ |  | Oleylamine | Oleic acid | Octadecene | 2H | - | X  (Only 2H-MoS_2_) | ChemCatChem 2024, 16, e202400264 |
| MoS_2_ | MoCl_5_ | Sulfur | Oleylamine | Octadecene |  | 1T` | - | X  (1T’-MoS_2_, MoSe_2_, WS_2_, WSe_2_) | J. Am. Chem. Soc. 2022, 144, 4863−4873 |
| MoS_2_ | Mo(CO)_6_ | Sulfur | Oleylamine | Oleic acid |  | 1T` & 2H | Through control of the S precursor reactivity | X  (MoS_2_, WS_2_)  Use of diverse precursors; limited generality | J. Mater. Chem. A, 2023, 11, 11341 |
|  |  | Sulfur | Oleic acid | ^1^TOP |  |  |  |  |  |
|  |  | ^2^TBDS | Oleylamine | Oleic acid |  |  |  |  |  |
|  |  | Dodecanethiol | Oleylamine | Oleic acid |  |  |  |  |  |
|  |  | Thiourea | Oleylamine | Oleic acid |  |  |  |  |  |
|  | MoCl_5_ | S-sualane | Oleic acid |  |  |  |  |  |  |
| MoS_2_ | MoCl_5_ | CS_2_ | Oleylamine | Oleic acid |  | 2H | - | X  (Only 2H-MoS_2_) | J. Phys. Chem. C 2019, 123, 10571−10577 |
| MoS_2_ | ^3^[Mo(Ac)_2_]_2_ | Thioacetamide | Oleylamine | ^4^TOPO | Squalne | 2H | - | X  (Only 2H-MoS_2_, WS_2_) | Nanoscale, 2016, 8, 15262–15272 |
| MoS_2_ | Mo(CO)_6_ | Dodecanethiol | Oleic acid | TOP |  | 2H | - | X  (2H-MoS_2_ , WSe_2_) | Small Methods 2021, 5, 2100558 |
| MoS_2_ | MoCl_5_ | Sulfur | Oleylamine | Oleic acid |  | 2H | - | X  (Only 2H-MoS_2_) | Small 2023, 19, 2207101 |
| MoSe_2_ | MoCl_5_ | Selenium | Oleylamine | Octadecene |  | 2H | - | X  (2H-MoS_2_, MoSe_2_) | ACS Appl. Nano Mater. 2022, 5, 10311−10320 |
| MoSe_2_ | MoO_2_(acac)_2_ | Selenium | Oleylamine |  |  | 2H | - | X  (Only 2H-MoSe_2_) | ACS Appl. Nano Mater. 2022, 5, 4293−4304 |
| MoSe_2_ | MoCl_5_ | ^5^(PhCH_2_)_2_Se_2_ | Oleylamine |  |  | 1T` & 2H | V  doping | X  (Only MoSe_2_) | ACS Nano 2021, 15, 14672−14682 |
| MoSe_2_ | MoCl_5_ | NaHSe | Oleylamine | Octadecene |  | 2H | - | X  (2H-MoS_2_, MoSe_2_) | ACS Appl. Mater. Interfaces 2024, 16, 36315 |
| MoSe_2_ | Na_2_Mo_4_ | Selnium | Oleic acid | Octylamine | Octadecene | 2H | - | X  (Only 2H-MoSe_2_) | Chem. Mater. 2015, 27, 3167−3175 |
| MoSe_2_ | Mo(CO)_6_ | Selenium | Oleic acid | - | Oleylamine | 2H | - | X  (Only 2H-MoSe_2_) | Electrochimica Acta 231 (2017) 69–76 |
| MoSe_2_ | MoCl_5_ | Ph_2_Se_2_ | Oleylamine | Octadecene | - | 1T` | - | X  (1T’-MoS_2_, MoSe_2_, WS_2_, WSe_2_) | J. Am. Chem. Soc. 2022, 144, 4863−4873 |
| MoSe_2_ | MoCl_5_ | Ph_2_Se_2_ | Oleylamine | Oleic acid | HMDS | 2H | - | X  (2H-MoSe_2_, WS_2_, WSe_2_) | J. Am. Chem. Soc. 2017, 139, 11096−11105 |
| MoSe_2_ | Mo(CO)_6_ | Ph_2_Se_2_ | Oleic acid | Oleylamine | Oleyl alcohol | 2H | - | X  (2H-MoSe_2_, WSe_2_) | J. Am. Chem. Soc. 2015, 137, 7266−7269 |
| MoSe_2_ | Mo(CO)_6_ | Selenourea | Oleylamine | - | - | 2H | - | X  (Only 2H-MoSe_2_) | Nanomaterials 2020, 10, 1786 |
| MoSe_2_ | H_2_MoO_4_ | Selenourea | Oleylamine |  |  | 2H | - | (Only 2H-MoSe_2_) | Nanomaterials 2020, 10, 1786 |
| MoSe_2_ | MoO_2_(acac)_2_ | (PhCH_2_)_2_Se_2_ | Oleylamine |  |  | 2H | - | X  (2H-MoSe_2_, WSe_2_ ) | Nanoscale, 2014, 6, 11046 |
| MoSe_2_ | Na2MoO4·2H2O, | Se-ODE | 1-octylamine | oleic acid | - | 2H | - | X  (2H-MoSe_2_, WSe_2_  Only 2H phase) | Nanoscale, 2017, 9, 13998–14005 |
| WS_2_ | W(CO)_6_ | CS_2_ | Ligand-free synthesis in Formamide  (Stabilized by inorganic ions, S²⁻) | | | 2H | - | X  (2H-MoS_2_, WS_2_) | ACS Appl. Mater. Interfaces 2020, 12, 13148−13155 |
| WS_2_ | W(CO)_6_ | (NH_4_)_2_S |  |  |  |  |  |  |  |
| WS_2_ | WCl_6_ | CS_2_ | Dioctylamine | Oleic acid | Octadecene | 1T` & 2H | Re  doping | X  (MoS_2_, WS_2_) | ACS Nano 2023, 17, 25731−25738 |
| WS_2_ | WCl_6_ | CS_2_ | Oleylamine | 1-Octadecanethiol | - | 1T` & 2H | Tuning the reactivity of the W precursor by ODT | X  (Only WS_2_) | 10.26434/chemrxiv-2023-tnggx |
|  |  |  |  | Octadecanethiol |  | 1T` |  |  |  |
|  |  |  |  | Dodecanethiol |  | 1T` |  |  |  |
|  |  |  |  | Hexadecanethiol |  | 1T` |  |  |  |
| WS_2_ | WCl_6_ | Sulfur | Oleylamine | Octadecene |  | 1T` | - | X  (1T’-MoS_2_, MoSe_2_, WS_2_, WSe_2_) | J. Am. Chem. Soc. 2022, 144, 4863−4873 |
| WS_2_ | WCl_6_ | CS_2_ | Oleylamine | Oleic acid | HMDS | 1T` & 2H | The reactivity of the W precursor by HMDS | X  (Only WS_2_) | J. Am. Chem. Soc. 2014, 136, 14121−14127 |
| WS_2_ | WCl_6_ | CS_2_ | Oleylamine | Oleic acid | HMDS | 2H | - | (2H-MoSe_2_ ,WS_2_, WSe_2_) | J. Am. Chem. Soc. 2017, 139, 11096−11105 |
| WS_2_ | W(CO)_6_ | Sulfur | Oleylamine | Oleic acid |  | 1T` & 2H | Through control of the S precursor reactivity | X  (MoS_2_, WS_2_)  Use of diverse precursors; limited generality | J. Mater. Chem. A, 2023, 11, 11341 |
|  |  | Sulfur | Oleic acid | TOP |  |  |  |  |  |
|  |  | TBDS | Oleylamine | Oleic acid |  |  |  |  |  |
|  |  | Dodecanethiol | Oleylamine | Oleic acid |  |  |  |  |  |
|  |  | Thiourea | Oleylamine | Oleic acid |  |  |  |  |  |
|  | WCl_6_ | S-sualane | Oleic acid |  |  |  |  |  |  |
| WS_2_ | WCl_6_ | Sulfur | Oleylamine | Oleic acid | HMDS | 2H | - | X  (Only 2H-WS_2_) | J. Phys. Chem. C 2021, 125, 18841−18848 |
| WS_2_ | (NH_4_)_2_WO_4_ | Thiourea | Oleylamine | Oleic acid |  | 1T` & 2H | Ligand | X  (Only WS_2_) | Nano Energy, 2018, 50, 176–181 |
| WS_2_ | W(CO)_6_ | Thioacetamide | Octadecanamine | TOPO | Squalne | 2H | - | X  (2H-MoS_2_, WS_2_) | Nanoscale, 2016, 8, 15262–15272 |
| WS_2_ | WCl_6_ | Sulfur | Oleylamine | Oleic acid | Octadecene | 1T` & 2H | reaction temperature | X  (Only WS_2_) | Nanoscale, 2022, 14, 15859–15868 |
| WS_2_ | H_2_WO_4_ | Thiourea | Oleylamine |  |  | 2H | - | X  (Only 2H-WS_2,_) | Processes 2021, 9, 1491 |
| WS_2_ | WCl_4_ | CS_2_ | Oleylamine |  |  | 2H | - | X  (Only 2H-WS_2_) | Sci. Rep. 2019, 9, 9002 |
| WS_2_ | WCl_6_ | CS_2_ | Oleylamine | Oleic acid | HMDS | 2H | - | X  (Only 2H-WS_2_) | Chem. Sci., 2019, 10, 10310–10317 |
| WSe_2_ | WCl_6_ | (PhCH_2_)_2_Se_2_ | Oleylamine |  |  | 2M & 2H | Temperature &  W:Se | X  (Only WSe_2_) | Adv. Mater. 2024, 36, 2307867 |
| WSe_2_ | W(CO)_6_ | Ph_2_Se_2_ | Oleic acid | TOPO |  | 1T` & 2H | Oleic acid/TOPO ratio and reaction time | X  (Only WSe_2_) | Chem. Commun., 2019, 55, 8856--8859 |
| WSe_2_ | W(CO)_6_ | Ph_2_Se_2_ | TOPO | Hexadecane |  | 2M & 2H | W:Se | X  (Only WSe_2_) | Chem. Mater. 2024, 36, 8834−8845 |
| WSe_2_ | W(CO)_6_ | Selenium | Oleylamine |  |  | 1T` | Ligand | X  (Only WSe_2_) | Cryst. Growth Des. 2021, 21, 1451−1460 |
|  |  |  | Oleic acid |  |  | 2H |  |  |  |
| WSe_2_ | WCl_6_ | Ph_2_Se_2_ | Oleylamine | Octadecene |  | 1T` | - | X  (1T’-MoS_2_, MoSe_2_, WS_2_, WSe_2_) | J. Am. Chem. Soc. 2022, 144, 4863−4873 |
| WSe_2_ | W(CO)_6_ | Ph_2_Se_2_ | TOPO |  |  | 2H | - | X  (Only 2H-WSe_2_) | J. Am. Chem. Soc. 2016, 138, 13253−13259 |
| WSe_2_ | WCl_6_ | Ph_2_Se_2_ | Oleylamine | Oleic acid | HMDS | 2H | - | (2H-MoSe_2_ ,WS_2_, WSe_2_) | J. Am. Chem. Soc. 2017, 139, 11096−11105 |
| WSe_2_ | W(CO)_6_ | Ph_2_Se_2_ | Oleic acid | oleylamine | oleyl alcohol | 2H | - | X  (2H-MoSe_2_, WSe_2_) | J. Am. Chem. Soc. 2015, 137, 7266−7269 |
| WSe_2_ | W(CO)_6_ | Selenium | Oleylamine |  |  | 1T` | Ligand | X  (Only WSe_2_) | Nanoscale, 2020, 12, 22307–22316 |
|  |  |  |  | Oleic acid |  | 2H |  |  |  |
| WSe_2_ | W(CO)_6_ | (PhCH_2_)_2_Se_2_ | Oleylamine |  |  | 2H | - | X  (2H-MoSe_2_, WSe_2_) | Nanoscale, 2014, 6, 11046 |
| WSe_2_ | W(CO)_6_ | TOPSe | Oleic acid |  |  | 1T` | - | X (Only 1T’-WSe_2_) | Nat Commun 2019, 10, 712 |
| WSe_2_ | W(CO)_6_ | Ph_2_Se_2_ | Oleic acid | TOP |  | 2H | - | X  (2H-MoS_2_ & WSe_2_) | Small Methods 2021, 5, 2100558 |

* Universality indicates whether both 2H and 1T′ phases were selectively synthesized across multiple TMD systems using a unified synthetic protocol.

^1^TOP : Trioctylphosphine

^2^TBDS : *tert*-butyl disulphide

^3^[Mo(Ac)_2_]_2_ : Molybdenum(II) acetate dimer

^4^TOPO : Trioctylphosphine oxide

^5^MoO_2_(acac)_2_ : Bis(acetylacetonato)dioxomolybdenum(VI)

^6^(PhCH_2_)_2_Se_2_ : Dibenzyl diselenide

^7^Ph_2_Se_2_ : diphenyl diselenide

**Figure S1.** **HAADF-STEM images of WSe_2_ synthesized with different precursor injection durations under identical precursor ratios ([M]:[X] = 1:2) and ligand conditions (V_OLAc_/V_Total_ = 0). Images correspond to samples obtained after a) 3 h and b) 5 h of precursor injection.**

**Figure S2. Low-magnification HAADF-STEM images of various 1T′-phase and 2H-phase TMD nanosheets. (a) 1T′-MoS_2_, (b) 1T′-MoSe_2_, (c) 1T′-WS_2_, and (d) 1T′-WSe_2_ synthesized under optimized OLAm/OLAc ligand conditions. (e) 2H-MoS_2_, (f) 2H-MoSe_2_, (g) 2H-WS_2_, and (h) 2H-WSe_2_ synthesized under optimized OLAm conditions.**

**Figure S3. Lateral size distribution histograms of TMD nanosheets corresponding to Figures 2a (a), 2c (b), 3a (c), 3b (d), 3c (e), and 3d (f). Each dataset includes >100 nanosheets analyzed from low-magnification HAADF-STEM images. Note that the histograms for 2H WS₂ and 2H WSe₂ are excluded from this analysis; these samples predominantly form heavily stacked, multilayered aggregates, precluding the accurate measurement of individual nanosheet lateral dimensions.**

**Table S2. Summary of the number of monolayers and multilayers in 1T′- and 2H-phase TMDs synthesized, based on contrast analysis of HAADF-STEM images**

| **TMDs** | **Monolayer (n_1_)** | **Multilayer (n_2_)** | **Percentage of Monolayer** |
| --- | --- | --- | --- |
| 1T` MoS_2_ | 86 | 0 | 100 % |
| 1T` MoSe_2_ | 80 | 4 | 95 % |
| 1T` WS_2_ | 92 | 5 | 95 % |
| 1T` WSe_2_ | 91 | 3 | 96 % |
| 2H MoS_2_ | 96 | 0 | 100 % |
| 2H MoSe_2_ | 80 | 4 | 95 % |
| 2H (mainly) WS_2_ | 2 | 42 | 4.5 % |
| 2H (mainly) WSe_2_ | - | 31 | 0 % |

**Figure S4. HAADF-STEM images of WS₂ and WSe₂ synthesized under intermediate precursor molar ratios, where mixed phases are observed. a) WS₂ synthesized at [W]:[S] = 1:32 and b) WSe₂ synthesized at [W]:[Se] = 1:4.**

**Figure S5. XPS spectra of metal core-level for phase-pure TMD. Top panels show the 1T′ phase, and bottom panels show the 2H phase for a) WS₂, b) WSe₂, c) MoS₂, and d) MoSe₂, respectively. The fitted components confirm the distinct chemical states associated with each crystalline phase.**

**Figure S6.** XPS spectra of chalcogen core-level for phase-pure TMD. Top panels correspond to the 1T′ phase, and bottom panels correspond to the 2H phase for a) WS₂, b) WSe₂, c) MoS₂, and d) MoSe₂, respectively. The fitted components confirm distinct chemical states associated with each crystalline phase.

**Figure S7.** Raman spectra of monolayer TMD nanosheets in the 1T’ and 2H phases. Raman scattering results are shown for a) WS_2_, b) WSe_2_, c) MoS_2_, and d) MoSe_2_, respectively. Dashed lines indicate characteristic Raman modes used to distinguish between the 1T′ and 2H phases..

**Figure S8. HAADF-STEM images of MoS₂ and MoSe₂ synthesized under intermediate ligand compositions, where both 1T` and 2H regions are observed within individual flakes. a) MoS_2_ synthesized at [M]:[S] = 1:5 with V_OLAc_/V_Total_ = 0.1 and b) MoSe₂ synthesized at [M]:[Se] = 1:2 with V_OLAc_/V_Total_ = 0.2.**

**Figure S9. HAADF-STEM images of W-based TMD synthesized under high OLAc content conditions. a) shows WS_2_ synthesized at 340 °C with [W]:[S] = 1:64 and V_OLAc_/V_Total_ = 0.3. b) shows WSe₂ synthesized at 340 °C with [W]:[Se] = 1:20 and V_OLAc_/V_Total_ = 0.3.**

**Figure S10. Polarization curves of the 1T`- MoS_2_ obtained at a scan rate of 5 mV s^-1^ before and after a 60 h stability test conducted at a constant current density of −100 mA cm^−2^.**

**Table S3. Comparison of HER performance of phase-controlled TMD catalysts in 0.5 M H_2_SO_4_ with representative catalysts from literature.^*^**

| **Material** | **Phase** | **Synthesis method** | **Conducting additive** | **Post-treatment** | **Overpotential (mV) at**  **10 mA cm^−2^** | | **Tafel slope**  **(mV dec^−1^)** | **Stability** | **Ref.** |
| --- | --- | --- | --- | --- | --- | --- | --- | --- | --- |
| 1T′-MoS_2_ QDs/CC | 1T′ | Colloidal synthesis | - | - | 193.5 | 61.4 | | 60 h at a constant current density of −100 mA cm^−2^ | This work |
| 2H-MoS_2_ QDs/CC | 2H | Colloidal synthesis | - | - | 233.1 | 117.3 | | - | This work |
| 1T′-WS_2_ QDs/CC | 1T′ | Colloidal synthesis | - | - | 308 | 110.2 | | - | This work |
| 2H-WS_2_ QDs/CC | 2H | Colloidal synthesis | - | - | 413.5 | 97.1 | | - | This work |
| MoS_2_ ND/rGO | 2H | Colloidal synthesis | rGO + Vulcan XC-72 carbon | Annealed at 800 °C for 2 h  under Ar | 222 | 59.8 | | 1,000 cycles (−0.3 to 0.0 V vs. RHE) | Nano Res. 2017, 10, 2210. |
| Monolayer MoS­_2_ | 1T′ | Colloidal synthesis | - | Treated with acetic acid | 149 | 42 | | 48 h at a constant potential of −0.165 V vs. RHE | J. Am. Chem. Soc. 2022, 144, 11, 4863–4873. |
| 1T′-D WS_2_ | 1T` | Colloidal synthesis |  | Treated with thioglycolic acid | 200 | 50.4 | | 12 h at a constant potential of −0.3 V vs. RHE | Nano Energy, 2018, 50, 176-181. |
| MoSe_2_ NSs | 2H | Colloidal synthesis |  | Annealed at 400 °C for 1 h under Ar | 207 | 56 | | 2,000 cycles (−0.2 to 0.0 V vs. RHE) | *ACS Nano* 2021, 15, 3, 5467–5477. |
| 2H-MoSe_2_ HDH | 2H | Hydrothermal synthesis | Ketjen black | - | 191 | 72 | | ~11 h at a constant potential of −0.245 V vs. RHE | Nano Res. 2017, 10, 2667. |
| MoS_2_ QDs | 2H | Hydrothermal synthesis | - | - | 300 | 59 | | ~2 h at a constant potential of −0.3 V vs. RHE | J. Mater. Chem. A 2015, 3, 10693. |
| MoS_2_ NPs | 2H | Solution phase synthesis | - | - | 112 | 72 | | 1,000 cycles (−0.2 to −0.1 V vs. RHE) | Adv. Funct. Mater. 2024, 34, 2404565. |
| MoS_2_ QDs | Mixed (2H-dominant) | Electrochemical exfoliation | - | - | 210 | 60 | | - | Chem. Commun. 2015, 51, 6293. |
| WS_2_ QDs/CFP | 2H | Electrochemical exfoliation | - | - | 190 | 90 | | ~18 h at a constant potential of −0.265 V vs. RHE | Catal. Sci. Technol. 2019, 9, 223. |
| 1T-MoS_2_ QDs | 1T (high fraction) | Electrochemical Li intercalation + liquid exfoliation | - | - | 92 | 44 | | 80 h at a constant current density of −200 mA cm^−2^ | ACS Nano 2018, 12, 308. |
| MoS_2_ QDs/NFs/FTO | 2H | Liquid-phase exfoliation | - | - | 250 | 74 | | 1,000 cycles (−0.6 to 0.0 V vs. RHE) | ACS Sustainable Chem. Eng. 2018, 6, 8374. |
| Graphene / 2H-MoS_2_ | 2H | Liquid-phase exfoliation | Graphene flakes | - | 136 | 141 | | ~3.3 h at a constant potential of −0.5 V vs. RHE | Chem. Mater. 2017, 29, 5782−5786. |
| WS_2_ Quantum Sheets | 2H | Ball milling + solvent exfoliation | Ketjen black | - | 285 | 116 | | 12 h at a constant current density of −10 mA cm^−2^ | Nanotechnology 2021, 32, 495701. |
| 2H-MoS_2_ QDs | 2H | Pulsed laser ablation in liquid (PLAL) of bulk MoS_2_ | - | - | 534 | 57 | | - | J. Appl. Phys. 2021, 129, 025112. |

**^*^**Note: Table includes only pristine, phase-controlled TMDs reported in earlier studies (pure 1T or 2H phases); heterostructured, alloyed, and doped TMD materials are excluded.
